# Metformin Modulates *Cyclin D1* and *P53* Expression to Inhibit Cell Proliferation and to Induce Apoptosis in Cervical Cancer Cell Lines

**DOI:** 10.31557/APJCP.2019.20.6.1667

**Published:** 2019

**Authors:** Ratih Dewi Yudhani, Indwiani Astuti, Mustofa Mustofa, Dono Indarto, Muthmainah Muthmainah

**Affiliations:** 1 *Departement of Pharmacology, *; 3 *Departement of Phisiology,*; 4 *Departement of Anatomy, Faculty of Medicine, Sebelas Maret University, Surakarta, *; 2 *Departement of Pharmacology, Faculty of Medicine, Gadjah Mada University, Yogyakarta, Indonesia. *

**Keywords:** Metformin, HeLa cell, cyclin D1, p53, apoptosis

## Abstract

**Background:**

Cervical cancer is one of the most prevalent gynecological cancers worldwide and contributes in high mortality of Indonesian women. The efficacy of chemotherapy as a standart therapy for cervical cancer decreases because it frequenly rises adverse effects. Recent studies have found that metformin has a potential anticancer effect mostly through reduction of cyclin expression and activation of Activated Adenosine Monophosphate Kinase (AMPK). This study aimed to investigate the effect of metfomin on expression of *cyclin D1* and *p53* and apoptosis in HeLa cancer cell line.

**Methods:**

HeLa cells were treated with various doses of metformin and doxorubicin as a positive control. Cytotoxic effect of metformin was determined using the MTT assay. Immunocytochemistry was used to assess *cyclin D1* and *p53* expression and apoptosis levels of treated HeLa cells were analyzed using flowcytometry. Data of *cyclin D1* expression was statistically analyzed using the Kruskal-Wallis test followed by the Tamhane test, whilst ANOVA and Tukey post Hoc tests were used to analyze data of *p53* and apoptosis level. The significant value was p< 0.05.

**Results:**

Metformin was able to inhibit proliferation of HeLa cells with IC50 60 mM. HeLa cells treated with 60 and 120 mM metformin had lower *cyclin D1* expression than HeLa cells treated without metformin and reached a significant difference (p= 0.001). Moreover, 30 mM or higher doses of metformin increase significantly *p53* expression (p< 0.001). Induction of apoptosis was observed in HeLa cells treated with all doses of metformin and reached statistically difference (p= 0.04 and p < 0.001).

**Conclusion:**

Metformin can modulate *cyclin D1* and *p53* expression in HeLa cancer cell line, leading to inhibition of cell proliferation and induction of apoptosis. Other cyclin family members, CDK inhibitors and AMPK signaling should be further investigated in order to know mechanism of metformin action.

## Introduction

Cervical cancer which is generally caused by Human Papilloma Virus (HPV) infection is the first prevalent gynecological cancers worldwide (Hutchinson and Klein, 2008; Torre et al., 2015). In 2012, new cases of cervical cancer were estimated 527,600 patients and it caused 265,700 deaths in the world (Torre et al., 2015). In Indonesia, cervical cancer is the most common malignancy and the leading cause of death among women for the last three decades (Azis, 2001; Tobing et al., 2014). However, recent data have indicated that cervical cancer becomes the second top rank of morbidity and mortality in Indonesia after breast cancer (WHO, 2014).

HeLa cell line is HPV type 18 infected-cervical cancer cells and widely used in various experimental studies (Scheffner et al., 1991; Watts and Denise, 2010). In HeLa cells, E6 HPV protein enhances degradation of p53 protein through binding with leucine rich motifs of E6-associated protein in the ubiquitin ligase (Scheffner et al., 1993; Saha et al., 2012; Ajay et al., 2012; Martinez-Zapien et al., 2016). These protein binding complexes contribute in cisplatin resistance of some cancer cells including cervical cancer (Florea and Büsselberg, 2011; Yang-Hartwich et al., 2014; Zhu et al., 2016). Thus, restoration of p53 function is crucial in the management of HPV-associated cervical cancer because HeLa cells were treated with celecoxib (non-steroidal anti-inflammatory drug) could re-activate p53 via induction of ataxia telangiectasia mutated-/p38 mitogen-activated protein kinase and down regulation of cycloxygenase-2 (Saha et al., 2012). 

Surgery, radiotherapy and or chemotherapy are commonly used for cervical cancer treatment but none of them provides a favorable outcome since most patients are late diagnosed. Chemotherapy agents, for instance, inhibit not only cancer cells but also high proliferative normal cells. Therefore, it can reduce the therapy efficacy and often causes serious adverse effects (Goldie, 2001; Fujimoto, 2009). Due to the weaknesses of the existing therapy, it has driven intensive research to discover and to develop new therapeutic strategy for the management of cervical cancer.

Metformin (N,N-dimethylbiguanide), which is used as the gold standard therapy of type 2 diabetes, has a potential anticancer effect (Correia et al., 2008; Quinn et al., 2013). Epidemiological studies have reported that diabetic patients who got metformin had lower risk for breast cancer than control patients (Bodmer et al., 2010; Bosco et al., 2011). Metformin is also able to inhibit cell proliferation of prostate, colon and lung cancers either in vitro or in vivo studies (Sahra et al., 2008; Iliopoulos et al., 2011; Luo et al., 2012). 

The inhibitory effects of metformin in cancer cells are mediated by some signaling pathways (Dowling et al., 2007; Sahra et al., 2008; Rocha et al., 2011; Sikka et al., 2012). The most common mechanisms are activation of AMPK and reduction of cyclins expression (Dowling et al., 2007). AMPK phosphorylation inhibits mammalian Target of Rapamycin (mTOR) and fatty acid synthesis, resulting in induction of cell cycle arrest via direct p53 phosphorylation (Jones et al., 2005; Micic et al., 2011). Activation of p53 at Ser 15 and 46 residues leads to p53 stabilization and accumulation in mitochondria, which initiate the intrinsic apoptotic pathway (Nieminen et al., 2013). Therefore, the aim of this study was to investigate the potential anticancer effect of metfomin in *cyclin D1* and p53 expression and apoptosis in HeLa cancer cell line.

## Materials and Methods

All chemicals used in this study were obtained from Invitrogen Carsblad, USA unless otherwise stated. Metformin and doxorubicin were purchased from Kalbe Farma, Jakarta, Indonesia. DMEM powder, fetal bovine serum (FBS), amphotericin B, penicillin-streptomycin, 0.25% (w/v) trypsin EDTA and phosphate Buffer Saline solution were purchased from Gibco®, Invitrogen. Whilst 4-(2-hydrocyethyl)-piperazine-ethane) sulphonic acid (HEPES) was from Sigma Aldrich, USA and bicarbonate sodium was from Nacalai Tesque. Sodium dodecyl sulphate and chloric acid were purchased from Merck, Germany whereas 3-(4,5-dimethylthiazol-2yl)-2,5-diphenyl tetrazolium bromide (MTT) was obtained from Bio Basic Inc^®^. Antibody anti human *cyclin D1* and p53 were obtained from Biocare^®^ and annexin V Fluos staining kit was from Roche^®^, USA. 

*Cells culture*

The HeLa cell line was obtained from stocks at Parasitology Laboratory, Faculty of Medicine, Gadjah Mada University, Yogyakarta, Indonesia. Cells were grown in DMEM suplemented with 10% FBS, 2% penicillin-streptomisin and 0.5% fungizone and were maintained at 37°C in a 5% CO_2_ incubator (Heraeus HERA cell). 

*Cytotoxicity assay*

After 2 x 10^4^ HeLa cells were incubated for 24 h, the cells were treated with a serial dose of metformin or doxorubicin. Treated cells were then incubated at 37ºC in a 5% CO_2_ incubator and cells were added with 0.05% (w/v) MTT after 6 hour time point. After that, cells were added with 20% (w/v) SDS in 20 mM HCl and incubated at room temperature for overnight before reading it a spectrophotometer (Bio-Rad, Hercules, USA) with optical density 595 nm. The percentage of viable cell was calculated using a formula, adopted from Cancer Chemoprevention Research Center / CCRC (CCRC., 2009a):

$\mathrm{\% cell viability}=\frac{\left(\mathrm{Absorbance of treated cells}–\mathrm{Blank}\right)}{\%(\mathrm{Absorbance of control cells}–\mathrm{Blank})}\times 100$

Inhibition concentration (IC_50_) of metformin or doxorubicin was determined by using probit analysis.

*Immunocytochemistry*

Expression of *cyclin D1* and *p53* protein was determined by using immunocytochemistry as previously described in CCRC (2009b), De Falco et al., (2004) and Maki (2010). 2 x 10^5^ HeLa cells were grown on to a coverslip in a 24 well plate for 24 h at 37^o^C and the following day cells were treated with 30, 60 or 120 mM metformin. After 24 h incubation, attached cells were fixed with cold methanol and then were permeabilized with 10% (v/v) H_2_O_2_ solution. Fixed cells were incubated with diluted antibodies anti human *cyclin D1* or *p53 *at 4^o^C for 24h. The excess primary antibodies were washed with phosphate buffered saline (PBS) and then incubated with biotin linked secondary antibodies at room temperature for 20 minutes. Streptavidin coupled with horse radish peroxidase (HRP) was added and bound to biotin-conjugated secondary antibodies. To generate brown cells, 100 µl diaminobenzidine solution was added and counterstained with Mayer’s hematoxilin and eosin. *Cyclin D1* and *p53* expression were quantified as the percentage of total cells in 5 fields of each slide from different dose treatments (Dougherty et al., 2011).

*Apoptosis analysis*

2 x 10^5^ HeLa cells were grown in a 6 well plate for 24 h at 37^o^C and the following day cells were treated with 30, 60 or 120 mM metformin. After 24 h incubation, treated cells were stainned with 100 µl fluoresceinisothiocyanate (FITC)-conjugated anexin V and propidium iodide in the buffer containing 10 mM Hepes/NaOH pH 7.4, 140 mM NaCl and 5 mM CaCl2 and were incubated in the dark at room at RT for 15 minutes. The proportion of apoptotic cells was measured using FACSCalibur (Becton-Dickinson, USA). 

*Statistical analysis*

Average of *cyclin D1* expression was statistically analyzed using a non parametric test, Kruskal-Wallis followed by Tamhane test because the variance of experimental data was not homogene. Meanwhile ANOVA test followed by Tukey post Hoc test were used to determine different effects of metformin treatment in HeLa cells, especially on p53 expression and apoptosis. The significance value was set up at P<0.05.

## Results

*Cytotoxic effect of metformin*

In this study, inhibition of cell proliferation was used to evaluate the cytotoxic property of metformin, compared with doxorubicin as a chemotherapy standard for cervical cancer treatment. Table 1 indicated that metformin and doxorubicin administrations reduce HeLa cell proliferation in dose dependent manner but higher concentration of metformin (approximately 60 mM) is required for inhibition of 50% cell growth than that of doxorubicin (15 µM). An initial inhibition of cell growth appeared in HeLa cells were treated with less than 10 mM metformin. The inhibition of cell growth doubled after administration of 20 and 40 mM metformin. In contrast to the metformin cytotoxic effect, an initial concentration of doxorubicin (5 µM) was able to inhibit 40% HeLa cell growth. However, the inhibition of cell growth is similar when HeLa cells were treated with 11, 22 or 43 µM doxorubicin respectivelly. 

*Cyclin D1 expression in HeLa cells treated with metformin*

**Figure 1:**
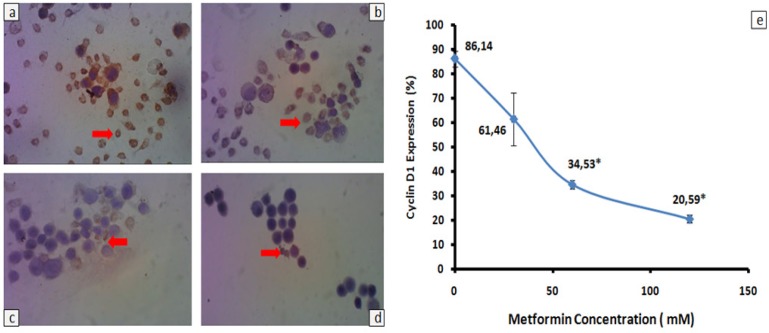
Cyclin D1 Expression in HeLa Cells Treated with Metformin. 2 x 10^5^ HeLa cells were grown on to a coverslip treated with (a) 0 mM, (b) 30 mM, (c) 60 mM, and (d) 120 mM metformin. Cells expressing cyclin D1 were analyzed by using immunocytochemistry with antibody anti human cyclin D1. Brown-stained cells (red arrows) were calculated from 5 point views with x400 magnification and all data were presented as percentage ± standard deviation (e). Experiments were performed in triplicate. *Significant value between treatmen and control group were set up at p < 0.05

*Cyclin D1* is one of cell cycle regulatory proteins that plays an important role in the control of mammalian cell proliferation (Matsushime et al., 1994). Therefore, we investigated whether or not metformin administration decreased *cyclin D1*expression in HeLa cells (Figure 1). Brown-stained cells decreased gradually in conjunction with increased doses of metformin after 24h incubation (Figure 1A-D). The lower percentage of *cyclin D1* expression was observed in HeLa cells treated with 30, 60 or 120 mM metformin, compared with HeLa cells treated without metformin (Figure 1E). However, decreased percentage of *cyclin D1* expression reached significant difference when the cells were treated higher than or equal with IC50 doses of metformin (p = 0.01).

*p53 expression in HeLa cells treated with metformin*

**Figure 2:**
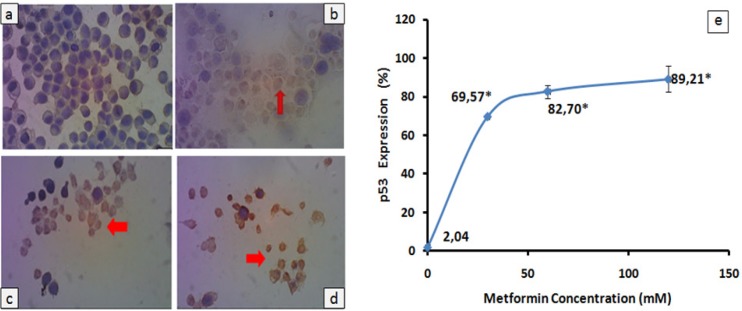
p53 Expression in HeLa Cells Treated with Metformin. 2 x 10^5^ HeLa cells were grown on to a coverslip treated with (a) 0 mM, (b) 30 mM, (c) 60 mM, and (d) 120 mM metformin. Cells expressing p53 were analyzed by using immunocytochemistry with antibody anti human p53. Brown-stained cells (red arrows) were calculated from 5 point views with x400 magnification and all data were presented as percentage ± standard deviation (e). Experiments were performed in triplicate. *Significant value between treatment and control group were set up at p < 0.05

The p53 protein is a transcription factor which is involved in cell cycle arrest, DNA repair and apoptosis (Amundson et al., 1998 ; Ghobrial et al., 2005). In HeLa cells, this protein is down regulated by an oncogenic E6 HPV protein (Scheffner et al., 1991 ; Saha et al., 2012). So, changed expression of the p53 protein in HeLa cells was evaluated after treatment with three doses of metformin (Figure 2). HeLa cells expressing p53 protein became brown-color after treatment with metformin (Figure 2b-d). After treatment with 30 mM metformin, p53 protein expression in HeLa cells sharply increased compared with negative control and it reached significant difference (p < 0.001). While slightly increased p53 expression was observed in HeLa cells treated with 60 or 120 mM metformin (82.70 or 89.21% respectivelly). This p53 expression was significantly different from negative control (p < 0.001).

*Metformin induces HeLa cell apoptosis*

**Figure 3:**
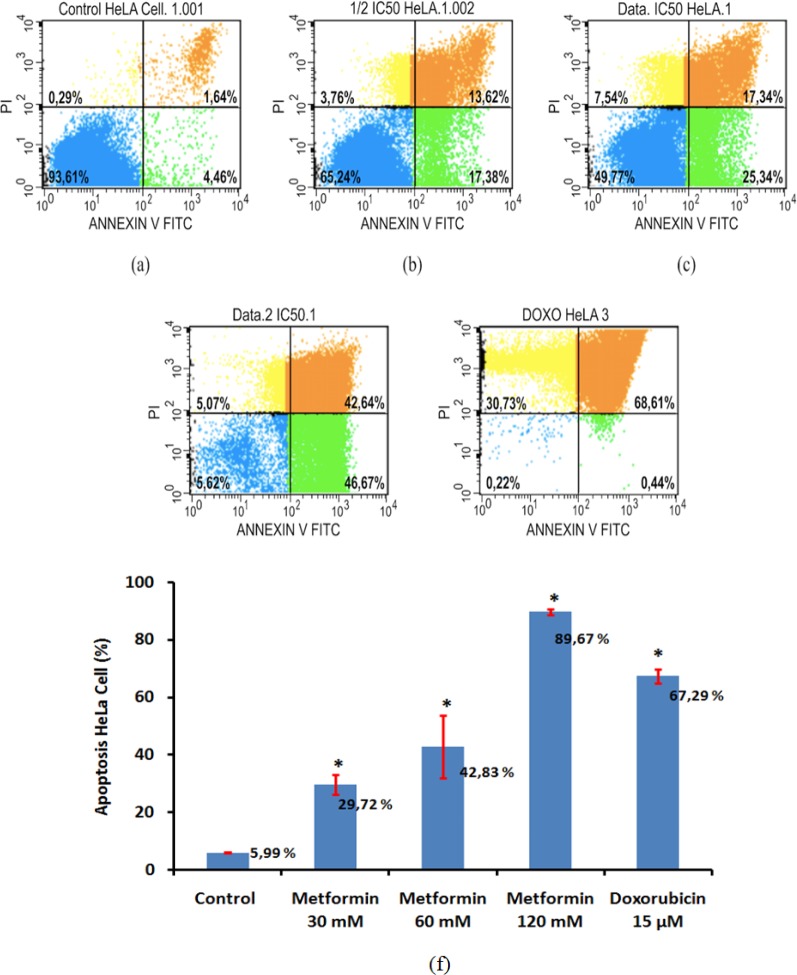
Apoptosis in HeLa cells treated with metformin. 2 x 10^5^ HeLa cells were incubated for 48 h in the presence of 30 mM (b), 60 mM (c) and 120 mM (d) metformin whilst cell only was a negative control (a) and 15 µM doxorubicin (e) was positive control. Apoptosis levels were analyzed using flow cytometry. Results represent three independent experiments (f). *Significant value between treatment and control group were set up at p < 0.05

Flow cytometry analysis using annexin V and propidium iodide staining was performed to examine apoptosis levels in HeLa cells in the presence of metformin or doxorubicin as a positive control. As can be seen in Figure 3b-d, metformin induced apoptosis in dose-dependent manner with the greatest effect observed in 120 mM. Apoptotic cells increased significantly in HeLa cells treated with 30 and 60 mM metformin, compare with HeLa cells without metformin treatment (p = 0.04 and p < 0.001 respectivelly). In addition, apoptosis effect of 120 mM metformin in HeLa cells was higher than that of 15 µM doxorubicin whereas 60 mM or less metformin administration has lower apoptosis effect, compared with 15 µM doxorubicin (Figure 3f).

## Discussion

Metformin significantly inhibits the growth of some cancer cells like breast, lung and hematologic cancers through the activation of AMPK with mammalian target of rapamycin (mTOR) or metabolic pathways (Zakikhani et al., 2006 ; Faubert et al., 2013). Teoritically, AMPK can be activated by a serine / threonine kinase, liver kinase β1 (LKB1) and Calmodulin dependent kinase kinases β (CAMKKβ) (Shaw et al., 2004 ; Hardie and Alessi., 2013). In cervical cancer cells, the inhibitory effect of metformin has not been fully understood (Dowling et al., 2007; Xiao et al., 2012). 

In this study, we found that metformin inhibits HeLa cell proliferation with IC_50_ at ± 60 mM (Table 1). Larsson et al. (2006) have suggested that sinthetic drugs with the IC_50_ < 10 μM are considered as a potential anticancer drug in vitro. Meanwhile, Niehr et al., (2011) have reported that cancer cell line is metformin-resistance if the IC_50_ is more than 20 mM. Therefore, our results indicate that HeLa cells might be resistant to metformin. It is likely caused by low phosphorylation of AMPK due to in the absence LKB1 expression (Zakikhani et al., 2006 ; Shaw et al., 2004), eventhough our study does not evaluate this protein. 

Despite IC50 that was obseved in HeLa cells treated with metformin in this study, higher than IC_50_ in Niehr study, this drug has an ability to inhibit cell proliferation in a dose dependent manner. This result suggests that metformin is potentially combine with the standard chemotherapies for cervical cancer treatment. In addition, previous studies have reported that combination of metformin and paclitaxel is more effective to reduce cell growth of lung and endometrial cancers, compared with administration of either paclitaxel or metformin alone (Rocha et al., 2011; Hanna et al., 2012). Our recent study has also showed that 30 mM metformin combined with 5 µM cisplatin has a better cytotoxic effect in HeLa cells than other combination doses (Yudhani et al., 2016). Results of in vivo study conducted by Iliopoulos et al., (2011) support the potential combination effect of metformin and chemotherapy drugs. Metformin markedly increases anticancer effects of doxorubicin, paclitaxel, and carboplatin in mouse models with breast, prostate, or lung cancers through inhibition of tumor growths and prevention of tumor relapse. 

In the present study, we have documented that metformin diminishes *cyclin D1* expression, replenishes p53 expression and induces apoptosis in HeLa cells (Figure 1-3). Reduction of *cyclin D1* expression is also found in nasopharyngeal cancer (C666-1), prostate cancer (LNCaP) and head and neck squamous cell carcinoma (FaDU and Detroit 562) incubated with 5-20 mM metformin for 72 h (Sahra et al., 2008; Zhao et al., 2011; Sikka et al., 2012). Another study shows that metformin plays an important role in global inhibition of protein translation, including *cyclin D1* protein (Sikka et al., 2012). In normal cells, *cyclin D1* functions as a checkpoint at G1/S phase transition (Matsushime et al., 1994). Therefore, administration of metformin probably inhibit proliferation of HeLa cells via disruption the cell cyle progression.

In our study, p53 expression increases in HeLa cells treated with all doses of metformin (Figure 2). Our results are inline with Dowling et al., (2007)’s works. Metformin treatment in breast cancer cells results in activation of AMPK and followed by p53 phosphorylation. Activated p53 will subsequently induce p21 expression, which down regulates cyclin-CDK complexes. Hence cell cycles will be disrupted (Xia et al., 2011). Similar to our finding, administration of 8 mM metformin in melanoma cancer cells also increases p53 expression (Janjetovic et al., 2011). In the other hand, inhibition of cell growth in some breast cancer cells which were treated with metformin is independent p53. This inhibition is mediated by reduction of *cyclin D1* expression, which promotes interaction between CDK inhibitors (p27Kip1 and p21Cip1) and cyclin dependent kinase (CDK) instead of c*yclin D1-CDK* binding complexes (Zhuang and Miskimins, 2008). Taken together our data may suggest that the inhibitory effect of metformin in HeLa cells seem to be associated with reduction of *cyclin D1* and activation of *p53* although protein analysis of phosphorylated *AMPK* and *p21* has not been performed yet in our study. 

**Table 1:** The Percentage of Proliferation Inhibition and IC_50_ of HeLa CellsTreated with Metformin

Treatment	Variation Dose	Proliferation inhibition rate (% ± SD)	IC_50_ ± SD
Metformin (mM)	80	53.87 ± 7.10	59.62±9.03
	40	48.95 ± 6.09	
	20	22.79 ± 6.79	
	10	11.76 ± 10.99	
	5	4.85 ± 11.25	
	2.5	8.59 ± 15.75	
Doxorubicin (µM)	172	84.09 ± 1.01	15±0.002
	86	75.66 ± 6.18	
	43	50.40 ± 2.72	
	22	56.08 ± 5.88	
	11	43.31 ± 5.32	
	5	40.37 ± 1.08	

We have demonstrated that metformin also induces apoptosis in HeLa cells. Perhaps, it is related to cell cycle arrest and activation of p53. However, some recent publications have reported that anti-apoptotic effect of metformin varies in some cancer cells (Sahra et al., 2008; Alimova et al., 2009; Janjetovic et al., 2011; Yasmeen et al., 2011, Lesan et al., 2014). Janjetovic et al., (2011) reported that metformin-treated melanoma cells undergo p53 dependent apoptosis. Sahra et al., (2010) also reported that activated p53 is required for apoptosis in prostate cancer cells after given a combination of metformin and 2 deoxyglucose. After treatment with metformin, ovarium cancer cell lines (OVCAR-3 and OVCAR-4) also undergo cell cycle arrest and apoptosis. However this cell cycle arrest occurs in the S and G2/M checkpoints and the apoptotic effect is mediated by activation of caspases 3/7 and reduction of Bcl-2 family proteins (Yasmeen et al., 2011). In contrast to our data, some studies show that metformin promotes cell cycle arrest at the G0/G1 phase without induction of apoptosis in prostate and breast cancer cells (Sahra et al., 2008; Alimova et al., 2009). The different effects of metformin in the some cancer cells are probably caused by different experimental conditions and properties of cancer cell lines (Yasmeen et al., 2011).

Although metformin administration can inhibit cell proliferation through suppresion of *cyclin D1*, we have not established yet how metformin administration disrupts cell cycle checkpoints. In addition, our results show that metformin-activated p53 could inhibit cell proliferation and induce apoptosis, but we do not know whether the p53 activation is AMPK dependent or uses other signalling pathways. 

In conclusion, our data collectively provide evidence that metformin can inhibit cell proliferation by down-regulation of *cyclin D1* and induce apoptosis by up-regulation of p53 in HeLa cancer cells. Further investigation is required to figure out whether or not other members of cyclin family and *CDK* inhibitors are involved in either the* G1*,* G2* or M checkpoints. Flow cytometry and Western blotting analysis are used to determine expression of *AMPK*, *p2*1 and *Bcl-2* family to unravell anti-proliferative and apoptotic effects of metformin in HeLa cancer cells. 

## Funding

This study was supported by master scholarship programme from Indonesian Ministry of Research, Technology and Higher Education.

## Conflict of Interest

All of authors declare no conflict of interest.
